# Colonization of C57BL/6 Mice by a Potential Probiotic *Bifidobacterium bifidum* Strain under Germ-Free and Specific Pathogen-Free Conditions and during Experimental Colitis

**DOI:** 10.1371/journal.pone.0139935

**Published:** 2015-10-06

**Authors:** Verena Grimm, Katarina Radulovic, Christian U. Riedel

**Affiliations:** Institute of Microbiology and Biotechnology, University of Ulm, 89068, Ulm, Germany; Robert Koch-Institute, GERMANY

## Abstract

The effects of at least some probiotics are restricted to live, metabolically active bacteria at their site of action. Colonization of and persistence in the gastrointestinal tract is thus contributing to the beneficial effects of these strains. In the present study, colonization of an anti-inflammatory *Bifidobacterium bifidum* strain was studied in C57BL/6J mice under germ-free (GF) and specific pathogen-free (SPF) conditions as well as during dextran sulfate sodium (DSS)-induced colitis. *B*. *bifidum* S17/pMGC was unable to stably colonize C57BL/6J mice under SPF conditions. Mono-association of GF mice by three doses on consecutive days led to long-term, stable detection of up to 10^9^ colony forming units (CFU) of *B*. *bifidum* S17/pMGC per g feces. This stable population was rapidly outcompeted upon transfer of mono-associated animals to SPF conditions. A *B*. *animalis* strain was isolated from the microbiota of these re-conventionalized mice. This *B*. *animalis* strain displayed significantly higher adhesion to murine CMT–93 intestinal epithelial cells (IECs) than to human Caco–2 IECs (*p* = 0.018). Conversely, *B*. *bifidum* S17/pMGC, i.e., a strain of human origin, adhered at significantly higher levels to human compared to murine IECs (*p* < 0.001). Disturbance of the gut ecology and induction of colitis by DSS-treatment did not promote colonization of the murine gastrointestinal tract (GIT) by *B*. *bifidum* S17/pMGC. Despite its poor colonization of the mouse GIT, *B*. *bifidum* S17/pMGC displayed a protective effect on DSS-induced colitis when administered as viable bacteria but not as UV-inactivated preparation. Collectively, these results suggest a selective disadvantage of *B*. *bifidum* S17/pMGC in the competition with the normal murine microbiota and an anti-inflammatory effect that requires live, metabolically active bacteria.

## Introduction

Bifidobacteria are Gram-positive, non-motile anaerobic bacteria belonging to the *Actinobacteria* phylum [1]. They are found in various ecological niches including food, sewage and oral cavities but the most important habitat of bifidobacteria is the gastrointestinal tract (GIT) of humans and animals [1]. Bifidobacteria are one of the predominant bacterial groups of the human colonic microbiota. In early infancy, they can make up to 95% of the fecal flora of breast-fed babies [2–5]. After weaning, due to the introduction of solid foods, and constant exposure to food-derived and environmental microorganisms, the relative abundance of bifidobacteria decreases but, after establishment of a adult microbiota, numbers remain relatively stable at 3–6% of all bacteria [6].

The adult microbiota is a highly stable ecosystem [7]. Yet the composition of this microbial consortium displays a remarkable interindividual diversity on the species level. At the same time, the relative abundance of the major phyla and metabolic capabilities are highly conserved across humans [8]. This suggests that the members of the gut microbiota are selected to form a stable consortium based on their metabolic functions. The redundancy in metabolic functions amongst the major phyla, however, allows for a certain flexibility in the individual makeup of the microbiota composition on the lower phylogenetic levels. Once established, the indigenous microbiota is highly resistant to colonization by ingested bacteria and prevents overgrowth of resident opportunistic pathogens present at low levels within the intestinal tract [9].

Alterations in the composition of the GIT microbiota have been observed in various diseases including antibiotic- and infection-associated diarrhea, necrotizing enterocolitis, and atopic and allergic disease [10,11]. This provides a rationale for the use of bifidobacteria and other mutualistic microbes of the GIT to maintain or restore a balanced microbiota. Additionally, the intestinal microbiota is pivotal in the development of the mucosal immune system of the GIT in early infancy. [12]. The predominance of bifidobacteria during this period suggests that they play an important role.

Bifidobacteria and other commensal bacteria are extensively used as probiotics, i.e. live microbial supplements, in functional foods e.g. to reduce cholesterol levels, improve lactose intolerance, alleviate constipation [11] and protect against infections with enteric pathogens [13–15]. A further promising target for probiotic treatment are inflammatory disorders of the GIT [11]. Inflammatory bowel diseases (IBDs) are a group of chronic gastrointestinal disorders characterized by relapsing and remitting inflammation of the GIT. IBDs are multifactorial diseases with genetic predisposition, environmental factors and the intestinal microbiota involved [16,17]. There are numerous experimental models for IBDs in small animals including spontaneous colitis in susceptible mouse strains, genetically modified animals, adoptive transfer models and chemically induced models of colitis [18,19]. One of the most frequently used models of chronic intestinal inflammation is DSS-induced colitis in mice and rats [19,20]. DSS-induced colitis is characterized by bloody diarrhea, ulcerations and heavy infiltration of inflammatory cells into the mucosa most probably as a consequence of disruption of the epithelial barrier by DSS [19]. Moreover, the intestinal microbiota displays prominent alterations during DSS-induced colitis [21].

Various strains of bifidobacteria have shown promising anti-inflammatory effects [22–25]. One of these strains is *B*. *bifidum* S17, which was initially isolated from the feces of a breast-fed infant [26]. *B*. *bifidum* S17 adheres at high levels to cultured intestinal epithelial cells (IEC) [27,28] and displays potent anti-inflammatory activity both *in vitro* [28,29] and in two murine models of colitis [28,30].

Adhesion to intestinal epithelial cells and/or mucus is discussed as a feature that supports for colonization and persistence of bifidobacteria in the GIT [31] and therefore is one of the selection criteria for probiotics. Cell surface components that promote colonization and adhesion to the intestinal epithelium include sortase-dependent [32] and type IVb tight adherence pili [33], exopolysaccharides [14] and lipoproteins [34,35]. In addition to their role in colonization and persistence, pili of bifidobacteria were also shown to modulate immune responses [32].

In this study, the ability of an anti-inflammatory and potential probiotic *B*. *bifidum* strain to colonize C57BL/6J mice was investigated under GF and SPF conditions and during DSS-induced colitis.

## Materials and Methods

### Bacterial strains and growth conditions

In this study, *B*. *bifidum* S17/pMGC [36] was used. Additionally, a *Bifidobacterium sp*. strain was isolated from a fecal sample of a C57BL/6J mouse kept at the animal facility at the University of Ulm. For this purpose, a fecal pellet was homogenized in 1 ml of PBS and serial dilutions were plated on MRSc agar containing 200 μg/ml mupirocin. A single colony was repeatedly re-streaked on MRSc agar to ensure clonality. Chromosomal DNA was isolated using a standard protocol and the 16S rRNA gene was amplified by PCR using universal primers 27f-Bif (5’-AGGGTTCGATTCTGGCTCAG–3’) and 1492r (5’- ACGGCTACCTTGTTACGACTT–3’) [37]. The PCR product was sequenced by a commercial service provider (Eurofins MWG GmbH, Ebersberg, Germany) and the obtained sequence analyzed by EzTaxon [38]. The closest match in the EzTaxon database was *B*. *animalis* subsp. *animalis* ATCC25527(T) with 99,29% similarity and 100% completeness. Additionally, a phylogenetic tree was calculated using CLC Workbench (Version 7.6.2; Qiagen) with the corresponding 16S rRNA gene sequences of a number of representative *Bifidobacterium sp*. including both *B*. *animalis* subspecies (S1 Fig). This suggests that the isolated strain TFZ-M24 belongs to the species *B*. *animalis* subsp. *animalis*.

Bifidobacteria were cultured anaerobically in Lactobacilli MRS medium (Difco) supplemented with 0.5 g/L L-cysteine (MRSc) at 37°C. Anaerobic conditions were achieved by cultivation in sealed jars using AnaeroGen sachets (Merck). For cultivation of *B*. *bifidum* S17/pMGC, MRSc medium was supplemented with 5 μg/ml chloramphenicol.

For experiments with dead bacteria, 10 ml of an overnight culture *B*. *bifidum* S17/pMGC were washed in PBS, poured into a petri dish and exposed to UV light (302 nm) for 10 min using a UV transilluminator. Efficient inactivation was confirmed by absence of growth of an aliquot plated onto MRSc agar.

### Adhesion assays

Adhesion to Caco–2 (ATCC^®^ HTB–37™) and CMT–93 (ATCC^®^ CCL–223™) was determined by classical plate counting essentially as described previously [35]. Caco–2 and CMT–93 cells were maintained in Dulbecco's Modified Eagle Medium (DMEM) supplemented with 10% (v/v) FCS, 1% (v/v) non-essential amino acids (NEAA), and 1% (v/v) penicillin-streptomycin solution. Cells were incubated in cell culture incubators at 37°C with 5% CO_2_. Medium was changed every two to three days and cells were subcultured according to supplier’s guidelines.

For experiments, cells were grown to confluent monolayers for 18–21 (Caco–2) or 5–6 (CMT–93) days. At this stage approximately 1×10^6^ cells were counted per well for both cell lines. One day before experiments, cell culture medium was changed to DMEM with 1% NEAA but without FCS and antibiotics to prevent bacterial clumping or killing during the adhesion assay. Bacteria were grown in MRSc medium overnight, washed once with PBS, and adjusted to 1×10^7^ colony forming units per ml (CFU/ml) in DMEM with 1% NEAA and 500 μl of this suspension were added to a well containing 1×10^6^ cells, i.e. a bacteria to cell of 5:1. Following an incubation of 1 h to allow adherence, unbound bacteria were removed by three washing steps with 1 ml DMEM. Cells were lysed adding 500 μl ice-cold ddH_2_O, debris was scraped off the bottom of the well, and the lysate was transferred to a sterile Eppendorf cup. Wells were rinsed with 500 μl ice-cold ddH_2_O and the washings were combined with the debris to give a total volume of 1 ml. Serial 10- fold dilutions in PBS were plated in spots of 10 μl on MRSc agar plates and incubated for 48 h to enumerate CFU of adherent bacteria. Adhesion was then calculated as percentage of the number of bacteria added to the wells, which was determined by spot plating of the bacterial suspension added to cells. Adhesion experiments were performed in three technical triplicates on three independent bacterial cultures and cell passages (biological replicates).

### Animals

All animal experiments were approved by the ethical committee for animal experimentation of the University of Ulm and the responsible legal authority at the Regierungspräsidium Tübingen (Baden-Württemberg, Germany). C57BL/6J mice were bred and kept at the animal facility at the University of Ulm on a 14h/10h light/dark cycle at 21°C and 50–55% humidity under specific pathogen-free (SPF) or germ-free (GF) conditions. GF mice were housed under sterile conditions in a germ-free isolator. The gnotobiotic state was controlled weekly by screening for viral, bacterial, and fungal contaminations according to the FELASA recommendations. Mice received a standard laboratory chow and water *ad libitum*, which was sterilized for GF animals. For experiments, 7–12 week old mice of both sexes were used.

### Colonization of mice with *B*. *bifidum* S17/pMGC

For colonization experiments, each mouse was inoculated by three consecutive daily doses of 2×10^9^ CFU in 20 μl of PBS of *B*. *bifidum* S17/pMGC using a micropipette tip placed immediately behind the incisors. For quantification of fecal carriage of *B*. *bifidum* S17/pMGC, fecal pellets of all mice (n = 5–6 animals per experiment) were collected at the indicated time points after inoculation, weighed, and homogenized in 1 ml of PBS by vigorous vortexing. For determination of bacterial counts in small intestine, caecum, colon, and mesenteric lymph nodes, mice were disinfected *post mortem* by topical application of alcohol and dissection was performed using sterile surgical instruments. MLNs and the entire GIT were dissected. The GIT was cut into the three main sections (small intestine, caecum, colon). Each section was opened separately and washed vigorously twice in 2.5 ml PBS to separate luminal content from the tissue itself. Washings were combined and used to determine CFUs in luminal content. Washed tissue sections and MLNs were homogenized in tissue strainers (100 μm, BD Biosciences) and homogenates were used to determine CFU of tissue-adherent bacteria. CFUs were determined by plating serial dilutions in PBS on selective agar (MRSc containing 5 μg/ml chloramphenicol). In case of fecal samples of SPF mice, 200 μg/ml mupirocin were added to reduce the microbial background. Agar plates were incubated anaerobically for 48 h at 37°C. Bifidobacterial counts were determined as CFU/g feces or CFU/organ in the total luminal content or entire tissue homogenate. The limit of detection using this method is 1×10^3^ CFU/g feces (or organ) and is represented in figures by the minimum of the Y-axis.

### DSS-induced murine model of colitis

Female C57BL/6J mice (n = 4–5 per group) at 6–8 weeks of age were given 2% dextran sulfate sodium (DSS; MP Biomedicals LLC, colitis grade; average molecular weight: 36000–50000) in their drinking water for five days. For treatment with bifidobacteria, mice received daily doses of 2×10^9^ CFU in 20 μl of PBS as described above starting 5 days prior to DSS administration and treatment was continued until one day after DSS administration was stopped. Control mice were treated with PBS as placebo. Fecal carriage of *B*. *bifidum* S17/pMGC and body weight were recorded for each animal throughout the experiments and weight calculated as percentage relative to the weight immediately before DSS treatment on day 0. Mice were sacrificed by cervical dislocation and their colon was dissected between the ileocaecal junction and rectum. Fecal matter was removed by vigorously rinsing the lumen several times with PBS, colonic length and weight were recorded, and colon weight/length ratios were calculated as a macroscopic marker of inflammation [39].

## Results

### *B*. *bifidum* S17/pMGC is not able to colonize C57BL/6J mice under SPF but under GF conditions

**Fig 1:**
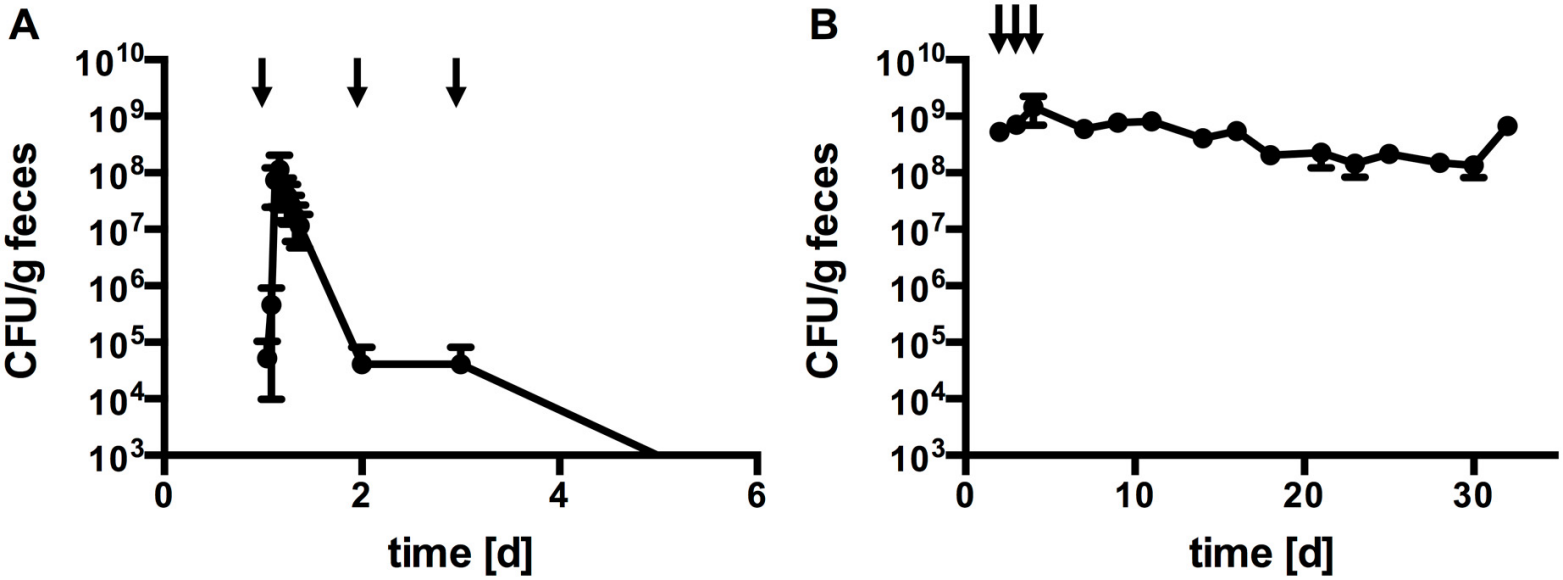
*B*. *bifidum* S17 is able to stably colonize GF but not SPF C57BL/6J mice. Fecal shedding of *B*. *bifidum* S17/pMGC following oral administration of three doses of 2×10^9^ CFU per animal on consecutive days (indicated as a black arrow) to C57BL/6J mice under SPF (A) or GF (B) conditions. Values are CFU/g feces and are mean ± standard error of the mean (*n* = 6 animals per experiment).

In a previous study, gastrointestinal transit of a *B*. *bifidum* S17 derivative was monitored in SPF mice [36]. As a next step and to extend on this initial experiment, it was investigated if a stable population of *B*. *bifidum* S17/pMGC could be established by administering the strain repeatedly on three consecutive days. The results of this experiment conforms previous findings in that fecal shedding peaked within 4–5 h after inoculation reaching approx. 1×10^8^ CFU/g feces and then constantly decreased to approx. 4×10^4^ CFU/g feces within 24 h, i.e. before administration of the next dose (Fig 1A, n = 6 animals). Similar low levels of *B*. *bifidum* S17/pMGC were detected 24 h after the second and third dose and bacterial shedding dropped below the limit of detection (i.e. 1×10^3^ CFU/g feces) 48 h after the last administration. These results indicate that *B*. *bifidum* S17/pMGC can not establish a stable population in mice harboring a normal SPF microbiota.

**Fig 2:**
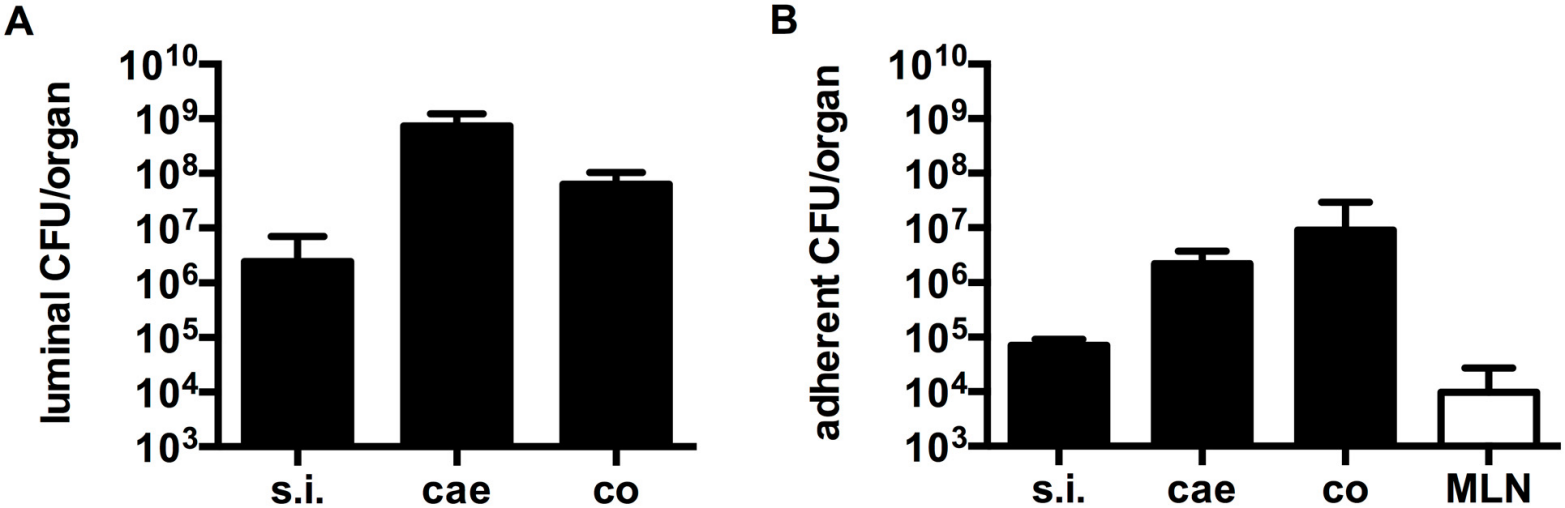
*B*. *bifidum* S17 is predominantly located in the caecum and colon of mono-associated C57BL/6J mice. Luminal and tissue-adherent counts of *B*. *bifidum* S17/pMGC in the small intestine (s.i.), caecum (cae), colon (co), and mesenteric lymph nodes (MLN) of mono-associated C57BL/6J mice for 32 days. Values are CFU/organ in the luminal content (A) or adherent to the tissue (B) of the different GIT sections or mesenteric lymph nodes (MLN) and are mean ± standard error of the mean (*n* = 6 animals per group).

To investigate if *B*. *bifidum* S17 is, in principle able to colonize the murine GIT and grow in this environment, the experiment was repeated using GF C57BL/6J mice. Following three daily doses, *B*. *bifidum* S17/pMGC was recovered at high levels (10^8^–10^9^ CFU/g feces) in fecal samples of mono-associated mice for more than 30 days (Fig 1B, n = 6 animals). Thus, in the absence of a normal microbiota, *B*. *bifidum* S17 is able to colonize the murine GIT. To obtain further information on the preferential colonization site of *B*. *bifidum* S17/pMGC in the murine GIT, different parts of the GIT as well as mesenteric lymph nodes of mono-associated mice were analyzed for the number of luminal (Fig 2A, n = 6 animals) and tissue-adherent bacteria (Fig 2B, n = 6 animals). All assayed sites harbored detectable levels of bacteria. Highest numbers of bacteria in the lumen were recorded in the caecum (7.4 ± 5.0 × 10^8^ CFU/organ). The picture slightly changed, when data was analyzed for total bacterial concentration in the luminal content. Highest concentrations were found in the colon (2.0 ± 0.9 × 10^9^ CFU/g luminal content; data not shown). The numbers of tissue-adherent bacteria paralleled the concentrations in the lumen with highest levels observed in the colon (Fig 2B). Interestingly, viable *B*. *bifidum* S17/pMGC could also be recovered from mesenteric lymph nodes of four out of six mice ranging from 9.6 × 10^3^ to 1.8 × 10^6^ CFU/g tissue (Fig 2B).

### *B*. *bifidum* S17/pMGC is unable to compete against a normal murine GIT microbiota

**Fig 3:**
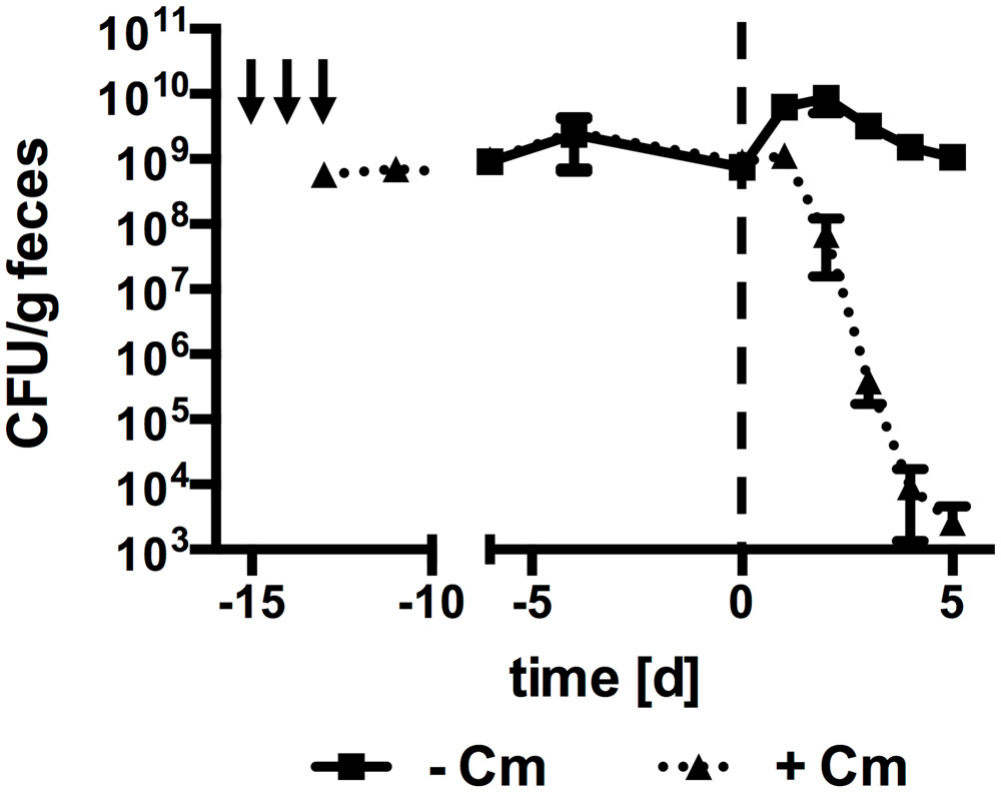
*B*. *bifidum* S17 is outcompeted from the GIT of mono-associated mice upon introduction of a normal microbiota. GF mice were mono-associated with *B*. *bifidum* S17/pMGC by three doses of 2×10^9^ CFU per animal on consecutive days (indicated as a black arrow) and maintained under GF conditions. After establishment of a stable population of *B*. *bifidum* S17/pMGC, mice were exposed to a normal microbiota by transfer to SPF conditions (day 0). Values are CFU/g feces and are mean ± standard error of the mean (*n* = 5 animals).

In a further attempt to establish a model system to investigate *B*. *bifidum* S17 and its effects in the presence of a normal gut microbiota, a stable population of *B*. *bifidum* S17/pMGC was established by mono-association of GF mice followed by transfer to SPF conditions (Fig 3, n = 5 animals). Upon transfer to SPF conditions, fecal counts of *B*. *bifidum* S17/pMGC rapidly dropped from 10^8^–10^9^ CFU/g feces by six orders of magnitude over the first six days and were below the limit of detection thereafter. At the same time, the number of colonies recovered from the same samples on MRSc agar containing mupirocin but without chloramphenicol remained constant throughout the entire experiment. This suggests, that *B*. *bifidum* S17/pMGC is gradually replaced by one or more mupirocin-resistant bacterial strains that are able to grow on MRSc, possibly bifidobacteria.

**Fig 4:**
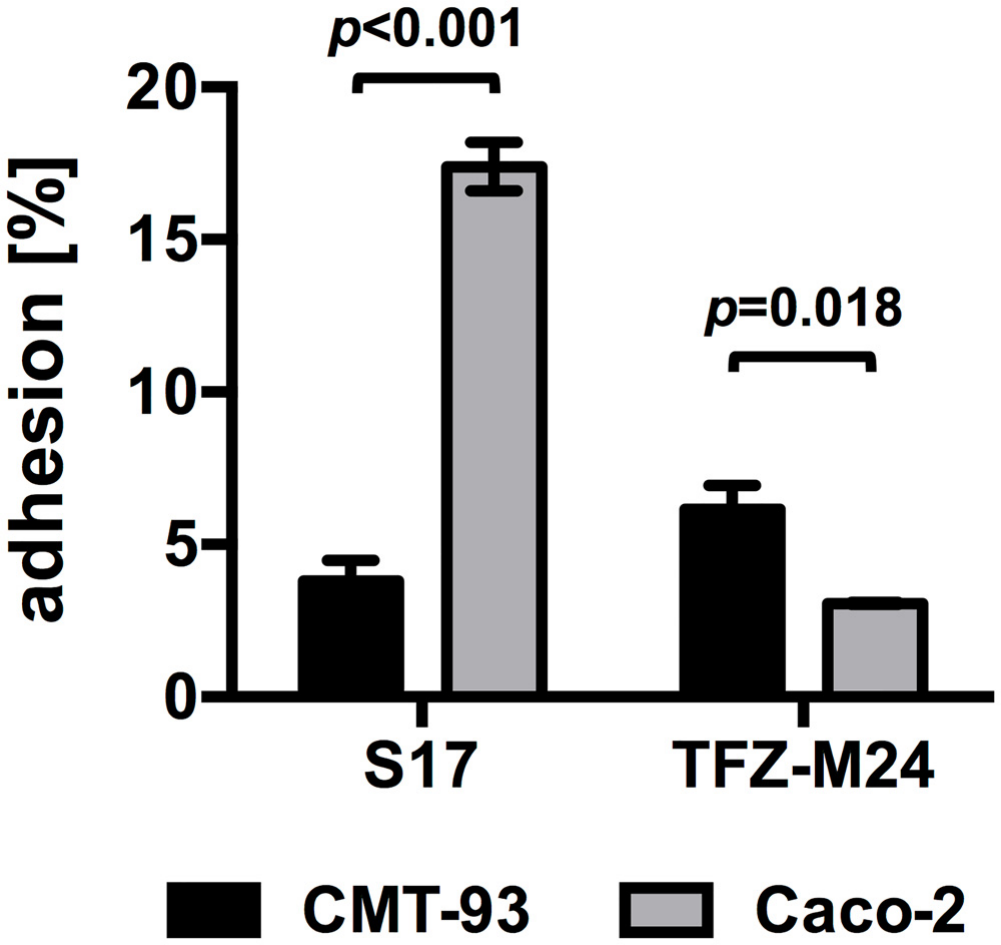
Host-specific adhesion of bifidobacteria to human and murine IECs. Adhesion of *B*. *bifidum* S17 and *B*. *animalis* TFZ-M24 to murine CMT–93 (black bar) and human Caco–2 (grey bars) IECs. Confluent cell monolayers were incubated with bifidobacteria at an MOI of 5 for 1 h and non-adherent bacteria were removed by washing. Amount of adherent adhesion is calculated as percentage relative to the initially added CFU. Values are mean ± standard deviation of three independent experiments performed in triplicate measurements. Statistical analysis was performed using Students *t*-test (*p*-values of the respective comparisons are indicated).

Experiments on GIT transit time of bifidobacteria indicated that mice in the animal facility at the University of Ulm harbor a background flora of bifidobacteria, which hampered the identification of markerless bifidobacteria on MRSc agar supplemented with mupirocin only [36]. Following up on these results, a bifidobacterial strain designated *B*. *animalis* TFZ-M24 was isolated from the feces of a C57BL/6J mouse housed in the animal facility. To test if host-specific differences in adhesion might be involved in the competitive exclusion of *B*. *bifidum* S17/pMGC adhesion experiments were performed using human and murine IEC lines. In line with previous studies [28,35], *B*. *bifidum* S17/pMGC showed significantly high adhesion to the human Caco–2 compared to murine CMT–93 IECS (Fig 4; p < 0.001, n = 3). By contrast, *B*. *animalis* TFZ-M24 adhered at significantly higher levels to the murine cell line CMT–93 compared to *B*. *bifidum* S17/pMGC (p = 0.018, n = 3). Similarly, *B*. *bifidum* S17 adhered better to human cells lines T84, and HT–29 than *B*. *animalis* TFZ-M24 (S2 Fig).

### Colonization and effects of *B*. *bifidum* S17/pMGC in DSS-induced murine colitis

**Fig 5:**
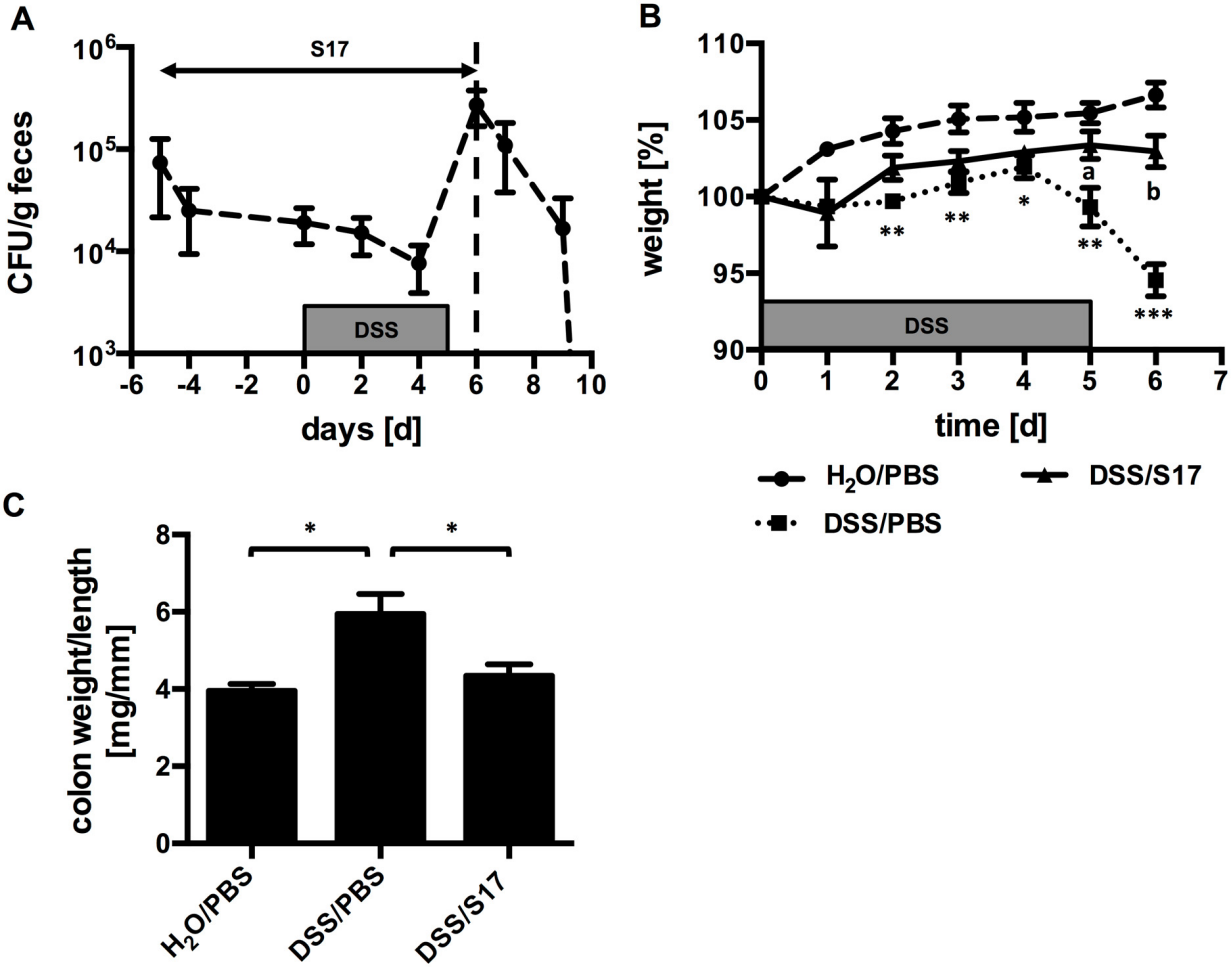
Colonization and effect of *B*. *bifidum* S17/pMGC in DSS-induced colitis. (A) Fecal counts of *B*. *bifidum* S17/pMGC in C57BL/6J mice before during and after administration of DSS. Animals received daily doses of 2×10^9^ CFU/animal of *B*. *bifidum* S17/pMGC starting 5 days prior to DSS challenge until to day 6 (i.e. 1 day after DSS treatment was stopped). Values are CFU/g feces and are mean ± standard deviation (*n* = 7 animals until day 6 and *n* = 3 thereafter). (B) and (C) Effect of *B*. *bifidum* S17/pMGC on DSS-induced weight loss (B) and colonic weight:length ratio (C). Mice of the DSS-challenged and *B*. *bifidum* S17-treated group (DSS/S17) are four out of seven animals shown in (A). Control mice received PBS as placebo and water with or without DSS (DSS/PBS and H_2_0/PBS respectively, both *n* = 4). Values are mean ± standard error of the mean. Statistical analysis was performed by one-way ANOVA with Bonferroni post-test analysis for each day (B) or at the end of the trial (C). Asterisks indicate levels of statistical significance differences for comparison to H_2_O/PBS group and letter for comparisons to DSS/PBS group (*: p < 0.05; **,^a^: p<0.01; ***,^b^: p<0.001).

To further investigate if changes in the microbiota or host physiology during colitis promotes colonization by *B*. *bifidum* S17/pMGC, C57BL/6J mice were pre-treated with *B*. *bifidum* S17/pMGC for 5 days and then administered DSS in drinking water for another 5 days to induce colitis. Treatment with bifidobacteria was continued until one day after DSS administration was stopped. Fecal shedding of *B*. *bifidum* S17/pMGC was monitored before, during and after DSS treatment (Fig 5A, n = 7 animals). This revealed that fecal levels of the strain were between 1 × 10^4^ and 1 × 10^5^ CFU/g during the pre-treatment and DSS phase of the experiment. However, numbers of fecal *B*. *bifidum* S17/pMGC quickly dropped and were below the limit of detection three days after treatment with bifidobacteria was stopped.

In parallel with the colonization experiment, two control groups received either DSS and a placebo treatment (PBS) or sterile H_2_O and placebo. DSS challenged, untreated mice showed considerable weight loss and were significantly different from the two other groups starting on day 1 into the DSS challenge until the end of the experiment (Fig 5B, n = 4–5 animals per group). Treatment of DSS-challenged animals with *B*. *bifidum* S17/pMGC prevented weight loss and this effect was statistically significant on days 5 and 6. To further analyze the effect of treatment with *B*. *bifidum* S17/pMGC, animals were sacrificed on day 6 and colons dissected. As a marker of inflammation colon weight:length ratios were calculated (Fig 5C, n = 4–5 animals per group). The group receiving placebo and DSS had a significantly higher colon weight:length ratio (5.9 ± 1.0 mg/mm) compared to the other groups. No difference could be observed between the control group receiving placebo and H_2_O (4.0 ± 0.3 mg/mm) and the DSS-challenged group receiving bacteria (4.3 ± 0.6 mg/mm).

**Fig 6:**
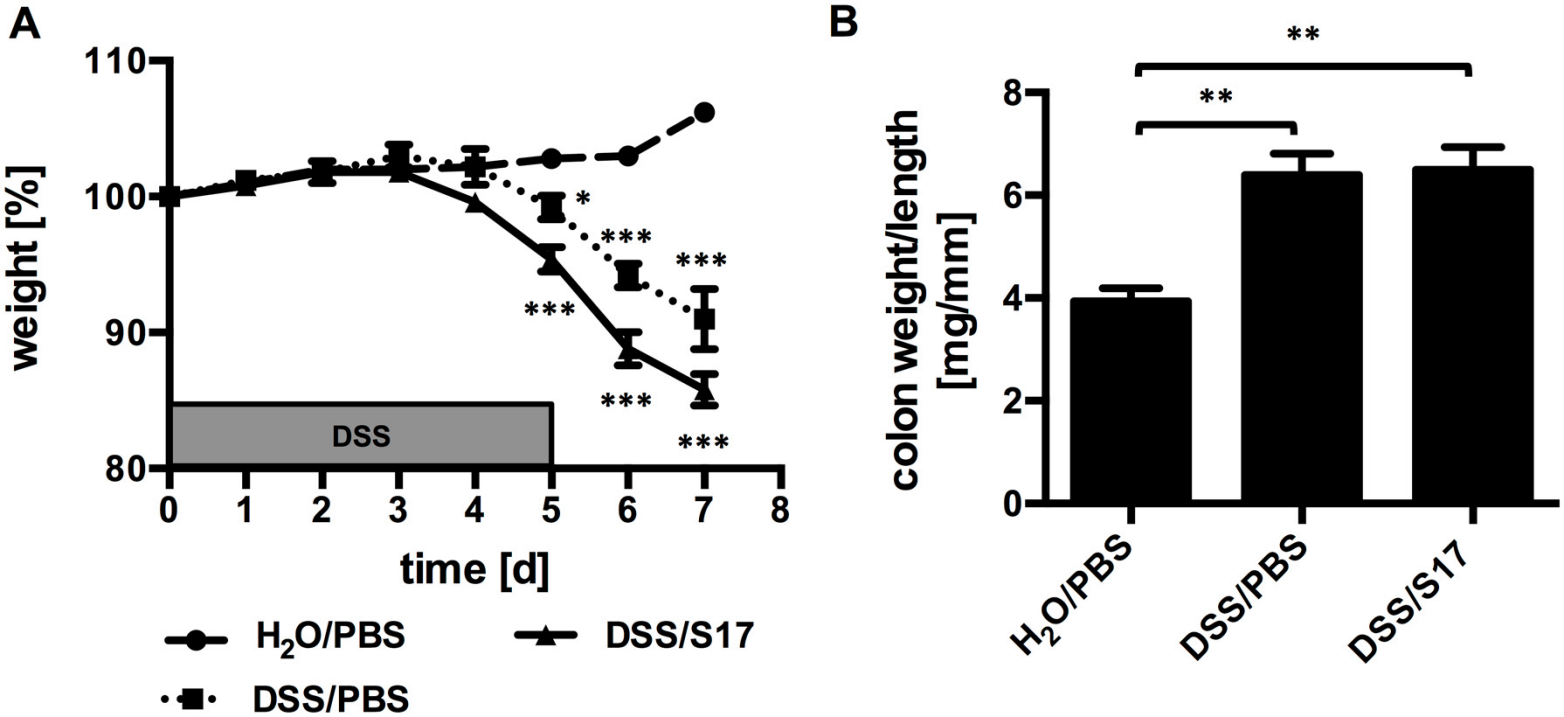
UV-killed *B*. *bifidum* S17 does not protect C57BL/6J mice against DSS-induced colitis. (A) Effect of UV-killed *B*. *bifidum* S17/pMGC on DSS-induced weight loss (A) and increase in colonic weight:length ratio (B). Mice were treated with *B*. *bifidum* S17/pMGC and challenged with DSS (DSS/S17). Control mice received PBS as placebo and water with or without DSS (DSS/PBS and H_2_O/PBS respectively, all groups *n* = 5). Values are mean ± standard error of the mean. Statistical analysis was performed by one-way ANOVA with Bonferroni post-test analysis for each day (B) or at the end of the trial (C). Asterisks indicate levels of statistical significance differences for comparison to H_2_O/PBS group (*: p < 0.05; ***: p<0.001).

Since stable colonization is not required for its probiotic effect, it was further investigated if administration of killed bacteria yields a similar protective effect. To this end, the DSS trial was repeated, however, mice were treated with UV-killed *B*. *bifidum* S17/pMGC. Inactivation of *B*. *bifidum* S17/pMGC completely abolished the protective affect on DSS-induced weight loss (Fig 6A). Also, the increase in colon weight:length ratio of DSS-challenged, placebo-treated animals compared to the mice receiving H_2_O and placebo (6.4 ± 0.9 vs. 3.9 ± 0.6 mg/mm) was not prevented by treatment with UV-killed bacteria (6.5 ± 1.0 mg/mm; Fig 6B).

## Discussion

Bifidobacteria are extensively used as probiotic supplements in functional foods. The present study was performed to gain insights into host colonization of *B*. *bifidum* S17, a strain that has shown promising anti-inflammatory activity *in vitro* and in two murine models of colitis [28–30]. Following oral administration to C57BL/6J mice under SPF conditions, fecal shedding peaked at 4–5 h post. This confirms previous studies on this and other *B*. *bifidum* strains [36,40] and is in good agreement with the physiological GIT transit time in mice [41]. Moreover, vast majority of *B*. *bifidum* S17/pMGC cells had passed through the GIT within 24 h and the strain was below the limit of detection 48 h after the last application clearly showing that it is unable to stably colonize SPF mice.

A slightly better colonization under SPF conditions was observed with *B*. *bifidum* PRL2010 in BALB/c mice [32,42]. In these studies, bacterial numbers gradually decreased and dropped below 10^5^ CFU/g feces 5 days after the last administration of *B*. *bifidum* PRL2010. Similarly, *B*. *adolescentis* L22 transiently colonized BALB/c mice at low levels but was 10^5^ CFU/g feces 4 days after the last administration [43]. Probably the best colonizer of SPF mice amongst bifidobacteria tested so far is *B*. *breve* UCC2003. For this strain, fecal carriage in SPF BALB/c mice was above 10^6^ CFU/g even 30 days after the last administration [14,33]. In all cited studies BALB/c mice were used that did not harbor detectable levels of bifidobacteria before administration of the tested strains. By contrast, experiments in the presented study were conducted with C57BL/6J mice that did harbor a considerable background of bifidobacteria [36]. BALB/c and C57BL/6J mice have a different genetic background and display largely different immune responses both under normal and pathological conditions [44–46] as well as following probiotic treatment [47]. The genetic background of the two mouse strains might therefore be, at least partially, responsible for the observed differences. Additionally, and maybe even more importantly, presence of indigenous bifidobacteria presumably poses a barrier for colonization by exogenous bifidobacteria.

Despite its inability to colonize mice with a normal microbiota, viable *B*. *bifidum* S17/pMGC were detectable in fecal samples of mono-associated mice at high levels (10^8^–10^9^ CFU/g feces) for at least 30 days after the last administration. This is in the range observed for gnotobiotic mice mono-associated with a number of other *Bifidobacterium sp*. strains [48,49]. Thus, colonization of mice by *B*. *bifidum* S17/pMGC is not limited by a general inability to survive and grow in the murine GIT. Moreover, the strain was also detected at relatively high numbers in the MLNs of monocolonized mice. Since this was observed more than 30 days after inoculation when a stable population is present in the GIT, this is probably not a consequence of high initial bacterial dosage but rather translocation or active sampling of luminal bacteria to lymphoid structures in the GIT. Fecal levels of *B*. *bifidum* S17/pMGC dropped rapidly to below the limit of detection upon transfer of mono-associated mice to an SPF environment. Similar observations were made for gnotobiotic Swiss-Webster mice associated with *Bacteroides thetaoitaomicron* and a *B*. *longum* subsp. *infantis* strain. When mice where bi-associated with both bacteria, the relative levels of *B*. *longum* subsp. *infantis* in the caecum were only 2% [50].

Another factor contributing to the inability of *B*. *bifidum* S17/pMGC to stably colonize mice might be a better adaptation to its original habitat, i.e. the human infant gut. Host adaptation of bifidobacteria has been shown on the level of carbohydrate utilization. For example, *B*. *longum* subsp. *infantis* strains are genetically adapted for utilization of human milk oligosaccharides (HMO) and are thus predominantly found in breast-fed, human infants [1,31,51]. Other species such as *B*. *longum* subsp. *longum*, *B*. *breve*, or *B*. *adolescentis* are deficient in HMO utilization but are equipped with the capacity to utilize plant oligo- and polysaccharides that are derived from the diet of the host. It would therefore not be surprising if other factors involved in host colonization such as adhesive structures and surface proteins would also display host specificity as shown for pathogenic bacteria [52].

The genome of *B*. *bifidum* S17 was shown to contain a large number of genes that might be involved in host colonization including Tad and sortase-dependent pili, lipoproteins, and several other genes encoding for surface proteins with domains known to mediate interaction with host structures [53]. For other bifidobacteria, some of these factors were already shown to contribute to adhesion to IECs and host colonization [31,54]. Compared to the murine isolate *B*. *animalis* TFZ–24, *B*. *bifidum* S17/pMGC adheres at high numbers to cultured human IEC lines Caco–2, T84 and HT–29 but adhesion was significantly lower to murine CMT–93 cells (Fig 4 and S1 Fig). These results indicate that bifidobacterial strains of human origin might be better adapted to the human environment and thus have a selective disadvantage in the murine GIT compared to murine bifidobacteria and other members of the murine gut microbiota.

Despite poor colonization and low fecal levels during colitis, *B*. *bifidum* S17/pMGC was able to reduce DSS-induced pathology as observed for *B*. *bifidum* S17 in TNBS-induced [30] and Rag-/- transfer colitis [28]. Similarly, other probiotic lactobacilli and bifidobacteria yielded positive effects in murine IBD models [20,24,25,55]. *B*. *bifidum* S17/pMGC was only effective in preventing signs of DSS-induced colitis when administered as live but not UV-killed bacteria. The question, whether or not viability of probiotics is a prerequisite for their effects is a matter of ongoing debate [56,57]. Depending on the application and mechanism of action, live and dead probiotic bacteria might be similarly effective or have different, maybe even divergent, effects. For example, a probiotic that inhibits pathogens by producing bacteriocins definitely needs to be viable. On the other hand, probiotics that act on the immune system via activation or inhibition of pattern recognition receptors such as Toll-like receptors (TLRs) might be similarly effective as a viable bacterium and as a UV-killed preparation since UV light does not destroy bacterial TLR ligands.

With respect to the anti-inflammatory mechanism of *B*. *bifidum* S17, it has to be kept in mind that sampling time for enumeration of fecal *B*. *bifidum* S17/pMGC was immediately prior to application of the daily dose of bifidobacteria. As shown by the results of the GIT transit time, *B*. *bifidum* S17 indeed is present at higher levels over the course of a day albeit not at constant levels. Thus, stable colonization is not necessary but a sufficient amount of live, metabolically active bacteria might be required for the protective effect of *B*. *bifidum* S17.

The mechanism by which *B*. *bifidum* S17 exerts its probiotic activity is still unknown. It may involve inhibition of excessive LPS-dependent NF-κB activation in IECs as shown previously for *B*. *bifidum* S17 *in vitro* [28,29]. Moreover, treatment with *B*. *bifidum* S17 may improve barrier function and/or induction of anti-inflammatory macrophage, dendritic, and T cell populations as shown for other bifidobacteria [23,25,58] or constitute a yet undescribed mechanism.

In either case, it remains to be seen in future experiments if the promising anti-inflammatory properties of *B*. *bifidum* S17 demonstrated in three murine models of colitis can be transferred to the human system. If the hypothesis that human bifidobacteria are better adapted to the human GIT is correct, this has important implications for studies on probiotic microorganisms. In this case, the mouse might not a suitable model system to study probiotics intended for humans. One possibility to overcome this limitation may be the use of humanized mouse strains once receptor and ligand of host and bacteria are well defined. Additionally, *in vitro* studies in primary human cells might more closely model the situation in humans than *in vivo* studies in mice. Nevertheless it can not be excluded that strains that demonstrate a positive effect in mouse models actually perform better in the human system due to a better adaptation to the microbial competition and nutritional conditions of the human GIT.

## Supporting Information

S1 Fig Taxonomic analysis of the *Bifidobacterium sp*. strain isolated from the animal facility at Ulm University. (PDF)

S2 Fig Adhesion of *B*. *bifidum* S17 and *B*. *animals* TFZ-M24 to cultured human IECs. (PDF)
