# Supplementary material for: Colonization of C57BL/6 Mice by a Potential Probiotic Bifidobacterium bifidum Strain under Germ-Free and Specific Pathogen-Free Conditions and during Experimental Colitis
Source: PLoS One. 2015 Oct 6;10(10):e0139935. doi: 10.1371/journal.pone.0139935 (PMC4595203; doi:10.1371/journal.pone.0139935)
Supplement: S1 Fig — (PDF) [file pone.0139935.s001.pdf]

S1 Figure

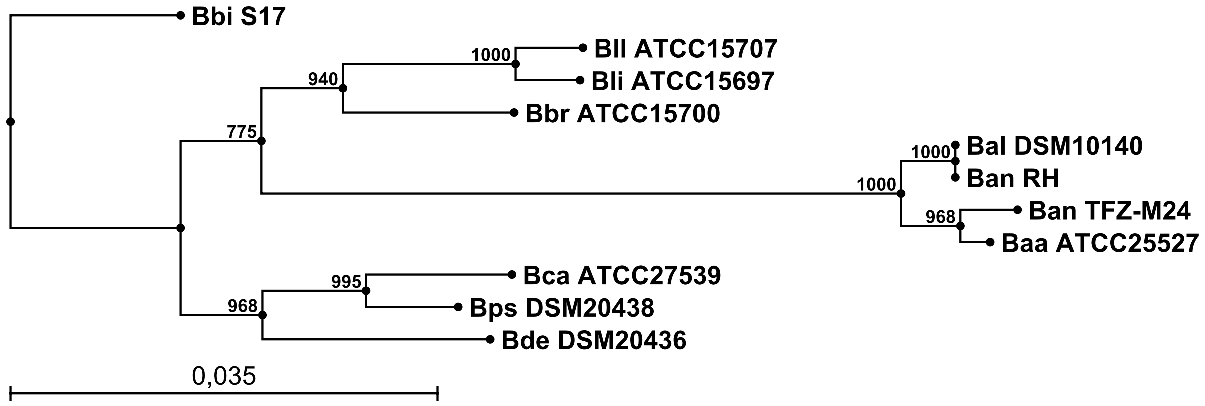

<u>Supplementary Figure S1:</u> **Taxonomic analysis of the *Bifidobacterium sp.* strain isolated from the animal facility at Ulm University.** A phylogenetic tree was calculated with the 16S rRNA gene sequence of the isolated strain (Ban TFZ-M24) obtained by PCR (described in Materials and Methods) and the corresponding sequences of *B. bifidum* S17 (Bbi S17), *B. longum* subsp. *longum* ATCC15707 (Bll ATCC15707), *B. longum* subsp. *infantis* ATCC15697 (Bli ATCC15697), *B. breve* ATCC15700 (Bbr ATCC15700), *B. animalis* subsp. *lactis* DSM10140 (Bal DSM10140), *B. animalis* RH (Ban RH), *B. animalis* subsp. *animalis* ATCC25527 (Bal ATCC25527), *B. catenulatum* ATCC27539 (Bca ATCC27539), *B. pseudocatenulatum* DSM20438 (Bps DSM20438), and *B. dentium* DSM20436 (Bde DSM20436). Numbers indicate bootstrap values from n=1000 iterations.
