# Supplementary material for: Colonization of C57BL/6 Mice by a Potential Probiotic Bifidobacterium bifidum Strain under Germ-Free and Specific Pathogen-Free Conditions and during Experimental Colitis
Source: PLoS One. 2015 Oct 6;10(10):e0139935. doi: 10.1371/journal.pone.0139935 (PMC4595203; doi:10.1371/journal.pone.0139935)
Supplement: S2 Fig — (PDF) [file pone.0139935.s002.pdf]

## S2 Figure

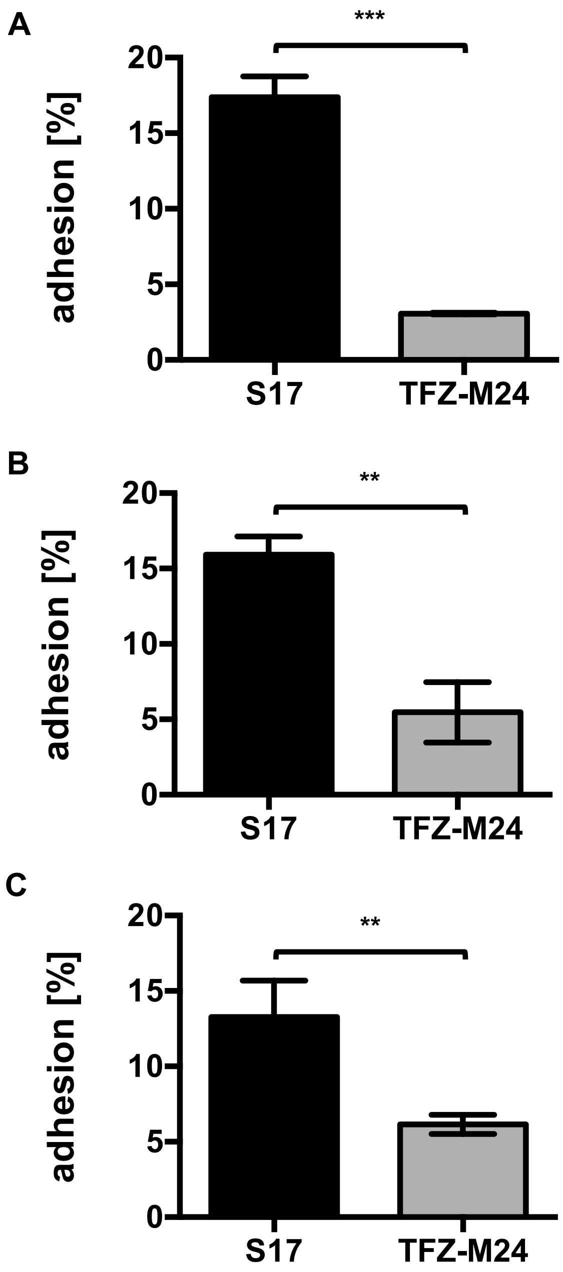


<u>Supplementary Figure S2:</u> **Adhesion of B. bifidum S17 and B. animals TFZ-M24 to cultured human IECs.** Adhesion of *B. bifidum* S17 and *B. animalis* TFZ-M24 to human Caco-2 (A), T84 (B), and HT-29 (C) cells. Confluent cell monolayers were incubated with bifidobacteria at an MOI of 5 for 1 h and non-adherent bacteria were removed by washing. Amount of adherent adhesion is calculated as percentage relative to the initially added CFU.

Values are mean ± standard deviation of three independent experiments performed in triplicate measurements. Statistical analysis was performed using Students *t*-test (**: $p < 0.01$; ***: $p < 0.001$).
